# SIRT1 Limits Adipocyte Hyperplasia through c-Myc Inhibition*

**DOI:** 10.1074/jbc.M115.675645

**Published:** 2015-12-11

**Authors:** Houari Abdesselem, Aisha Madani, Ahmad Hani, Muna Al-Noubi, Neha Goswami, Hisham Ben Hamidane, Anja M. Billing, Jennifer Pasquier, Michael S. Bonkowski, Najeeb Halabi, Rajaa Dalloul, Mohamed Z. Sheriff, Nasrin Mesaeli, Mohamed ElRayess, David A. Sinclair, Johannes Graumann, Nayef A. Mazloum

**Affiliations:** From the Departments of ‡ Microbiology and Immunology,; § Biochemistry, and; ¶ Genetic Medicine, Weill Cornell Medicine Qatar, Qatar Foundation, Education City, P.O. Box 24144, Doha, Qatar,; the ‖ Department of Genetics, Harvard Medical School, Boston, Massachusetts 02115, and; the ** Life Sciences Research Division, Anti-Doping Lab Qatar, P.O. Box 27775, Doha, Qatar

**Keywords:** adipogenesis, cell proliferation, hyperplasia, Myc (c-Myc), sirtuin 1 (SIRT1)

## Abstract

The expansion of fat mass in the obese state is due to increased adipocyte hypertrophy and hyperplasia. The molecular mechanism that drives adipocyte hyperplasia remains unknown. The NAD^+^-dependent protein deacetylase sirtuin 1 (SIRT1), a key regulator of mammalian metabolism, maintains proper metabolic functions in many tissues, counteracting obesity. Here we report that differentiated adipocytes are hyperplastic when SIRT1 is knocked down stably in mouse 3T3-L1 preadipocytes. This phenotype is associated with dysregulated adipocyte metabolism and enhanced inflammation. We also demonstrate that SIRT1 is a key regulator of proliferation in preadipocytes. Quantitative proteomics reveal that the c-Myc pathway is altered to drive enhanced proliferation in SIRT1-silenced 3T3-L1 cells. Moreover, c-Myc is hyperacetylated, levels of p27 are reduced, and cyclin-dependent kinase 2 (CDK2) is activated upon SIRT1 reduction. Remarkably, differentiating SIRT1-silenced preadipocytes exhibit enhanced mitotic clonal expansion accompanied by reduced levels of p27 as well as elevated levels of CCAAT/enhancer-binding protein β (C/EBPβ) and c-Myc, which is also hyperacetylated. c-Myc activation and enhanced proliferation phenotype are also found to be SIRT1-dependent in proliferating mouse embryonic fibroblasts and differentiating human SW872 preadipocytes. Reducing both SIRT1 and c-Myc expression in 3T3-L1 cells simultaneously does not induce the adipocyte hyperplasia phenotype, confirming that SIRT1 controls adipocyte hyperplasia through c-Myc regulation. A better understanding of the molecular mechanisms of adipocyte hyperplasia will open new avenues toward understanding obesity.

## Introduction

Obesity constitutes a major health problem worldwide (1–3). Excessive caloric intake, a sedentary lifestyle, and possible genetic predispositions are the main drivers of this epidemic (4–8). White adipose tissue (WAT)^2^ plays a central role in the development of obesity-associated comorbidities (5, 9). Under normal conditions, WAT serves several functions, such as storing energy in the form of fat, providing insulation to vital organs, and participating in immune responses and hormonal secretions (10). The expansion of WAT mass observed in the obese state is due to adipocyte hypertrophy and hyperplasia (4–8). The buildup of hypertrophic and dysfunctional adipocytes is the result of an imbalance of caloric intake *versus* expenditure (5, 9). Consequently, the functions of metabolic pathways in enlarged adipocytes are dysregulated (11, 12). The NAD^+^-dependent deacetylase SIRT1 has been shown to maintain proper metabolic functions in many tissues to protect against obesity (13). Recently, it has been demonstrated that a high-fat diet triggers inflammation-induced SIRT1 cleavage and inactivation in the adipose tissue of mice and promotes metabolic dysfunction (14). Mice engineered to overexpress SIRT1 or mice that were treated with small-molecule activators of SIRT1, such as resveratrol, were protected from high-fat diet-induced liver steatosis and insulin resistance (15–18). SIRT1 inhibits adipogenesis by repressing the transcriptional activity of peroxisome proliferator-activated receptor γ PPARγ (19). Adipose tissue-specific SIRT1 deletion in mice led to increased adiposity and metabolic dysregulation, including insulin resistance (14).

Knowledge regarding the mechanism that drives hyperplasia of adipocytes in the obese state is still lacking. Here we propose a novel function for SIRT1 in regulating this process. Our data show that SIRT1-silenced mouse 3T3-L1 preadipocytes differentiate into hyperplastic adipocytes. We show that these adipocytes are small, dysfunctional, and inflamed, as indicated by an increase in the gene expression of WAT and inflammatory markers and a decrease in brown adipose tissue markers. Interestingly, silencing of SIRT1 leads to enhanced proliferation potential in mouse 3T3-L1 preadipocytes. Using quantitative proteomics analysis, we demonstrate that the c-Myc pathway is altered, driving the enhanced proliferation phenotype in SIRT1-silenced preadipocytes. Follow-up studies in these cells reveal that c-Myc is hyperacetylated and activated, p27 protein levels are reduced, and CDK2 total and phosphorylated protein levels are increased. Moreover, differentiating SIRT1-silenced preadipocytes show enhanced MCE potential, which is accompanied by reduced p27, increased C/EBPβ, and increased c-Myc expression levels as well as hyperacetylated and activated c-Myc. We confirm SIRT1 dependence of c-Myc activation in 3T3-L1 cells and other preadipocyte cell models when SIRT1 signaling is ablated. The enhanced proliferation phenotype is also validated in proliferating *sirt1* knockout MEFs and differentiating SIRT1-silenced human SW872 preadipocytes. We also show that the Sirt1 knockdown-induced hyperplasia phenotype does not develop when c-Myc levels are reduced. We propose a model for adipocyte hyperplasia and dysfunction driven by the SIRT1/c-Myc pathway.

## Experimental Procedures

### 

#### 

##### Cell Lines/Cell Culturing

Murine 3T3-L1 cells were obtained as passage 8 (Zen-Bio) and used for experiments between passages 10 and 14. Cells were maintained in high-glucose DMEM (Invitrogen) supplemented with 10% calf serum, l-glutamine, penicillin, and streptomycin. 293T viral packaging cells (ATCC) were maintained in high-glucose DMEM supplemented with 10% fetal bovine serum, l-glutamine, and penicillin and streptomycin antibiotics. Human SW872 preadipocytes (ATCC) were cultured in DMEM/F12 medium supplemented with 8% calf serum, 15 mm Hepes, and penicillin and streptomycin antibiotics.

WT Mouse embryonic fibroblast (MEF) cells were isolated from 14-day-old mouse embryos and transformed as described earlier (20). Transformed *sirt1* knockout MEFs (a gift from Dr. Michael McBurney, University of Ottawa) were cultured in high-glucose DMEM supplemented with 15% FBS (Invitrogen) and penicillin and streptomycin antibiotics. All cells were grown at 37 °C in 5% CO_2_. The medium was changed every 2–3 days until cells achieved 70–80% confluence.

##### RNA Silencing and Generation of Lentiviral Particles

Stable lentiviral particles expressing shRNA targeting mouse SIRT1 mRNA, mouse c-Myc mRNA in 3T3-L1 preadipocytes, and human SIRT1 mRNA in SW872 preadipocytes were generated using a cDNA lentiviral shRNA vector (MISSION® shRNA plasmid DNA, Sigma-Aldrich). The respective sequences were as follows: shSirt1 (puromycin), 5′-CCGGAGTGAGACCAGTAGCATAATCTCGAGATTAGTGCTACTGGTCTCA CTTTTTTG-3′; shSirt1^(2nd Construct)^ (neomycin), 5′-CCGGGAGGGTAATCAATACCTGTTTCTCGAGAAACAGGTATTGATTACCCTCTTTTTG-3′; shMyc1 (neomycin), 5′-CCGGTGGAGATGATGACCGAGTTACTCGAGGTAACTCGGTCATCATCTCCATTTTTG-3′; shMyc1^(2nd Const.)^ (puromycin), 5′-CCGGGACTCCGTACAGCCCTATTTCCTCG AGGAATAGGGCTGTACGGAGTCTTTTTG-3′; and H-shSirt1(puromycin), 5′-CCGGGCGGCTTGATGGTAATCAGTACTCGAGTACTGATTACCATCAAGCCGCTTTTT-3′. We used a scramble non-sense RNAi sequence with no homology in the mouse or human genome (shScramble) as a control for the unspecific effects of shRNA (21). In brief, 293T cells were co-transfected with shRNA lentiviral plasmid or shScramble lentiviral plasmid plus the lentiviral packaging and envelope plasmids (21) using Lipofectamine 2000 (Invitrogen) according to the instructions of the manufacturer. The medium containing generated viral particles was collected 3 days after transfection. Generated shSirt1 lentiviral particles were used to infect 3T3-L1 and SW872 preadipocytes using 4 μg/ml Polybrene to generate stable shSirt1-expressing cells. Puromycin and G418 selections (2 μg/ml and 1 mg/ml, respectively) were used to select infected cells.

##### Adipocyte Differentiation

100% confluent murine 3T3-L1 cells were left for an additional 48 h and then induced to differentiate using a medium containing differentiation mixture (DMEM, 10% FBS, 1 μm dexamethasone (Sigma), 0.5 μm of methylisobutylxanthine (Sigma), and 10 μg/ml insulin (Sigma)). 2 days after differentiation induction, the medium was replaced with a maintenance medium containing DMEM, 10% FBS, and 10 μg/ml insulin and was changed every 2 days. SW872 preadipocytes were cultured up to 100% confluence and then induced to differentiate by adding oleic acid at a final concentration of 100 μm (22).

##### Oil Red O and LipidTOX Staining

The formation of lipid droplets was observed by phase-contrast microscopy (Zeiss) after staining with Oil Red O. In brief, cells were rinsed with PBS and then fixed with 4% paraformaldehyde for 15 min at room temperature. Cells were washed with PBS, rinsed with 60% isopropanol/PBS for 1 min, and then stained with Oil Red O (Sigma-Aldrich) for 1 h. Cells were then rinsed with 60% isopropanol/PBS for 30 s, followed by 3 consecutive washes with water. Nuclei were stained with hematoxylin for 6 min and then washed four times with water. Cells were mounted in 70% glycerol and then imaged using a digital EVOS^TM^ microscope equipped with an AMG camera. For LipidTOX staining of lipid droplets, cells were fixed with 4% paraformaldehyde for 30 min at room temperature. After one washing step in PBS, cells were stained with LipidTOX green (Invitrogen, diluted 1:1000). Sample preparations were then analyzed by flow cytometry (BD LSRFortessa) using BD FACS DIVA version 7.0 as the program software.

##### Growth Curve

3T3-L1 cells, MEFs, and SW872 preadipocytes (50 × 10^3^, 100 × 10^3^, and 400 × 10^3^ cells/well, respectively) were plated in 6-well plates. At different post-plating time points, the cell numbers were recorded using a TC10 automated cell counter (Bio-Rad).

##### Luciferase Reporter Assay

To monitor the transcriptional activity of c-Myc, we used the luciferase reporter construct (pMyc-TA-Luc, where TA is TATA box) that contained the c-Myc-specific response element (enhancer element), a TA promoter, and a luciferase reporter gene (Clontech). To determine the background level of reporter gene activity, we used a negative control construct, pTA-Luc, that lacks the enhancer element but contains a promoter and reporter gene (Clontech). As a control for transfection efficiency, we co-transfected all cells with the pSV-β-galactosidase plasmid. In brief, 80% confluent cells were transiently co-transfected with either pSV-β-gal and pMyc-TA-luc or pSV-β-gal and pTA-luc at a 1:2 ratio using Lipofectamine 2000 transfection reagent as directed by the protocol of the manufacturer (Invitrogen). 48 h after transfection, cells were lysed, and the luciferase assay was carried out using the Bright Glo luciferase assay system (Promega) according to the instructions of the manufacturer. The β-galactosidase assay was performed as described in Ref. 20. The firefly luciferase activity was normalized to β-galactosidase activity and expressed as relative luciferase activity.

##### BrdU Incorporation Assay and Cell Cycle Analysis

3T3-L1 cells (80–90% confluence) were pulsed for 6 h with 20 μm BrdU (Sigma). After fixation with 70% ethanol, detection of incorporated BrdU and cellular DNA content was performed as described in Ref. 23. In brief, cells were permeabilized in 0.1% Triton X-100 and 0.5% BSA, and then DNA was denatured by HCl (2 m) for 20 min at room temperature and washed with phosphate buffer. Cells were then stained with APC-conjugated antibodies against BrdU (BD Pharmingen^TM^, APC BrdU kit) for 1 h and counterstained with 7-aminoactinomycin D nuclear dye (Life Technologies). For cell cycle analysis, fixed 3T3-L1 cells were stained with 50 μg/ml propidium iodide (Life Technologies) and incubated with 0.1 μg/μl RNase A (Invitrogen) for 40 min at 37 °C in the dark. Sample preparations of BrdU and cell cycle assays were analyzed by flow cytometry (BD LSRFortessa) using BD FACS DIVA version 7.0 as the program software.

##### Western Blotting

Cells were lysed in radioimmune precipitation assay buffer (150 mm NaCl, 50 mm Tris-HCl (pH 7.5), 0.1% SDS, 1 mm EDTA, 1 mm EGTA, 0.5% sodium deoxycholate (Sigma), and 1% Triton X-100) supplemented with 1× protease inhibitor mixture (Sigma), 10 mm sodium fluoride (Sigma), 1 mm sodium orthovanadate (Sigma), 1 mm PMSF, 5 mm benzamidine (Sigma), 20 μg/ml calpain inhibitor (Sigma), 5 mm nicotinamide (Sigma), and 3 mm trichostatin A (InvivoGen). They were homogenized using a sonicator cell disrupter and centrifuged for 10 min at 4 °C and 10,000 × *g*. 15–30 μg of whole-cell extracts per sample was subjected to SDS-PAGE, followed by electroblotting onto PVDF membranes (Millipore) and Western blot analysis. Western blots were probed with the following antibodies (Abcam): mouse anti-SIRT1, rabbit anti-p27, mouse anti-PPARγ, rabbit anti-CDK2, rabbit anti-phospho-CDK2, rabbit anti-CEBPβ, rabbit anti-PGC1-α, rabbit anti-c-Myc, and rabbit anti-acetylated c-Myc (Lys-323) (Millipore). Quantitation of Western blot bands was performed using ImageJ software by selecting each band area, integrating the mean intensity and pixel value, and then dividing the product by the standard band, which was either actin or GAPDH. Relative intensity was then normalized with the control treatment or the initial time point as 1.

##### Quantitative RT-PCR

Total RNA was prepared with the RNeasy mini prep kit (Qiagen). First-strand cDNA was synthesized using the cDNA synthesis kit (Roche) following the instructions of the manufacturer. The cDNA was subjected to quantitative RT-PCR using a Quant Studio 6Flex thermal cycler (Applied Biosystems). Exon-exon junction TaqMan primers were used to amplify the studied genes (Applied Biosystems) (Adiponectin, Mm00456425_m1; Pank3, Mm00461298_m1; Chemerin, Mm00503579_m1; Agt, Mm00599662_m1; Fabp4, Mm00445878_m1; Adipsin, Mm01143935_g1; PRDM16, Mm00712556_m1; TNF-α: Mm00443258_m1; and IL10, Mm00439614_m1). Target gene expression was normalized to the endogenous control (actin, peptidylpropyl isomerase A, or GAPDH). The average Ct value from each triplicate was used to calculate -fold induction of the gene with the control group normalized to 1.

##### High-throughput Proteomics Analysis and Statistics

Quantitative proteomics using stable isotope labeling by amino acids in cell culture (SILAC) in conjunction with mass spectrometry on protein lysates isolated from 3T3-L1 cells was employed as described in Refs. 24, 25, followed by Ingenuity pathway analysis (IPA, Ingenuity Systems). Briefly, to obtain SILAC-labeled preadipocytes, cells were cultured in lysine- and arginine-free medium augmented with lysine and arginine of isotopic composition (“heavy”) with ^6^C_13_^2^N_15_ lysine (Lys-8) and ^6^C_13_^4^N_15_ arginine (Arg-10) for five population doublings, which yielded over 95% incorporation efficiency. After SILAC labeling of preadipocytes, equal protein amounts of labeled protein lysates were mixed with the experimental condition lysates to generate mixtures of heavy and light (unlabeled) lysates for quantitative proteomics. The experimental conditions included three biological replicates of shSirt1, shScramble, and uninfected 3T3-L1 lysates. shSirt1, shScramble, and uninfected 3T3-L1 preadipocytes grown to 60–80% confluence were harvested and lysed in lysis buffer (50 mm Tris-HCl (pH 8), 2% SDS, 1× protease inhibitors, 2 μl/ml benzonase, 2 mm sodium orthovanadate, 25 mm sodium fluoride, 5 mm nicotinamide, and 3 mm trichostatin A). Protein preparations were obtained after sonication and centrifugation. Equal amounts of protein of each of the replicates (100 μg) were mixed with SILAC-labeled lysates (100 μg). 200 μg of mixed protein lysates were precipitated by methanol/chloroform and subsequently subjected to in-solution digestion, followed by peptide separation using in-solution isoelectric focusing (12 fractions) as described in Ref. 25. Mass spectrometric analysis was performed, and the data were processed by the MaxQuant suite of algorithms (v. 1.3.0.5 (26, 27); UniProtKB *Mus musculus* was downloaded on August 20, 2013) using the parameters described in Ref. 25. Peptide identifications were transferred between aligned chromatographic features (“match between runs”) in MaxQuant using a 2-min retention time window. Statistically significant proteome changes upon comparisons of biological triplicates of uninfected, shScramble, and shSirt1–3T3-L1 proteomes were identified using Limma (28), a supplemental package to R (R Core Team 2013). To further characterize the proteome changes between conditions, we carried out pairwise *t* tests (two-tailed, unequal variance) between the three conditions (uninfected *versus* shScramble, uninfected *versus* shSirt1, and shScramble *versus* shSirt1) and adjusted the *t* test *p* values using the Benjamini-Hochberg procedure. The significance threshold was set at an adjusted *p* value of less than 0.05. In cases where quantification was available for only one of the three replicates, the values for the other two replicates were imputed using the standard deviation calculated from the standard deviation of the other conditions and the available expression value. Imputed values were expression (+) standard deviation and expression (−) standard deviation. Proteins with only one replicate in all conditions (where no variance could be calculated for any conditions) were not considered in subsequent statistical analyses.

## Results

### 

#### 

##### SIRT1 Reduction Leads to Hyperplastic, Small, Dysfunctional, and Inflamed Adipocytes

**FIGURE 1.**
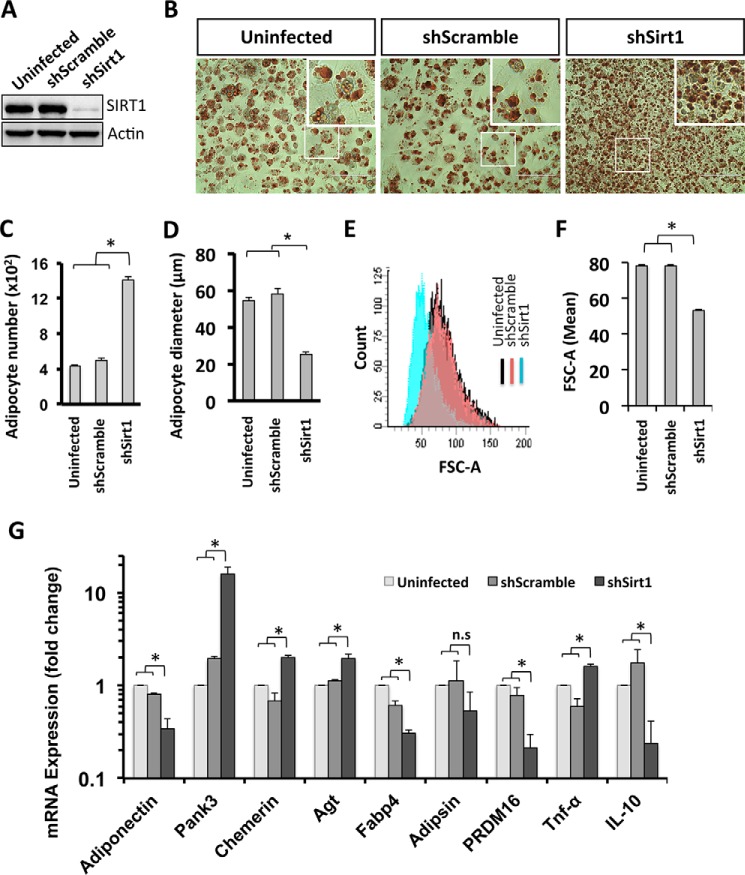
**Adipocytes are hyperplastic, small, dysfunctional, and inflamed when SIRT1 is silenced.** *A*, Western blotting was performed using antibodies against SIRT1 for the indicated conditions of 3T3-L1 cells (uninfected cells, shScramble, and shSirt1 lentivirus-infected cells). Actin was used as a loading control. *B*, Oil Red O staining of differentiated 3T3L-1 adipocytes for the indicated conditions. The *areas in boxes* are enlarged 5-fold at the *top right. Scale bars* = 100 μm. *C*, quantification of the data in *B* for the number of adipocytes for each condition. Results are presented as mean ± S.E. from six different fields of three independent experiments using Student's *t* test. *, *p* < 0.05. *D*, cell diameter calculation was quantified from adipocytes of the indicated conditions. Results are presented as mean ± S.E. from eight different fields of three independent experiments using Student's *t* test. *, *p* < 0.05. *E* and *F*, cell size estimation of 3T3-L1 adipocytes of the indicated conditions by flow cytometric analysis using LipidTOX staining. *E*, on the FITC+ gate, the cell size was estimated by FSC-A (forward scatter) (*blue curve*, shSirt1; *red curve*, shScramble; *black curve*, uninfected) and quantified as mean ± S.E. of three independent experiments using Student's *t* test. *F*, *, *p* < 0.05. *G*, mRNA expression analysis for white and brown adipocyte markers, and inflammation markers were assessed by quantitative RT-PCR analysis on d10PDI of the indicated conditions of 3T3-L1 adipocytes. Results are represented as mean ± S.E. of three independent experiments using Student's *t* test. *, *p* < 0.05; *n.s.*, not significant.

Here we investigated the function of SIRT1 in adipocyte differentiation by reducing SIRT1 expression. We developed a shRNA lentiviral vector targeting SIRT1 mRNA that allows for stable expression of shRNA-targeting SIRT1 (shSirt1) in 3T3-L1 preadipocytes. Using Western blot analysis, we demonstrated that the infection of 3T3-L1 preadipocytes with the lentiviral construct containing shSirt1 results in undetectable levels of the SIRT1 protein compared with control cells (uninfected and shScramble-expressing 3T3-L1 cells) (Fig. 1*A*). To evaluate whether SIRT1 knockdown affects adipogenesis in 3T3-L1 cells, preadipocytes were induced to differentiate, and mature adipocytes were stained with Oil Red O on day 6 post-differentiation induction (d6PDI) (Fig. 1*B*). We found that differentiated SIRT1-silenced cells were significantly higher in number than control cells (1400 ± 39 adipocytes/mm^2^ in shSirt1 *versus* 400 ± 12 adipocytes/mm^2^ and 450 ± 36 adipocytes/mm^2^ in uninfected and shScramble, respectively) (Fig. 1, *B* and *C*). We then characterized the effect of SIRT1 silencing on the metabolic function of terminally differentiated adipocytes by measuring adipocyte size and evaluating the expression levels of transcripts associated with mitochondrial biogenesis, WAT, and brown adipose tissue. The hyperplastic adipocytes in the SIRT1 knockdown lines were smaller than the control adipocytes (24- ± 1.06-μm diameter of SIRT1-silenced adipocytes *versus* 58- ± 3.07-μm and 53- ± 1.52-μm diameter of shScrambled and uninfected adipocytes, respectively) (Fig. 1*D*). To further confirm the difference in adipocyte cell size under SIRT1-silenced conditions compared with control cells, we measured the cell size of LipidTOX-labeled adipocytes using flow cytometric analysis. LipidTOX-FITC-unlabeled adipocytes were excluded, and only LipidTOX-FITC+ adipocytes were gated to measure adipocyte size with FSC-A scatter (Fig. 1*E*). Accordingly, the FSC-A mean of LipidTOX-labeled adipocytes was reduced significantly in SIRT1-silenced cells (53.5 ± 0.5 FSC-A) compared with control shScrambled and uninfected cells (78.5 ± 0.5 FSC-A and 78.5 ± 0.5 FSC-A, respectively), as shown in Fig. 1*F*. These data show that a loss of SIRT1 protein causes the generation of small adipocytes.

It is known that SIRT1 controls the gene expression of adiponectin via FoxO1-C, a hormone secreted by WAT to modulate metabolic processes (29). Here we analyzed adiponectin expression under SIRT1 knockdown conditions and found it to be down-regulated in mature adipocytes on day 10 PDI (Fig. 1*G*). To gain insights into how the SIRT1 level affects the physiological role of adipocytes, the expression levels of white and brown adipocyte markers were assessed (Fig. 1*G*). In SIRT1 knockdown adipocytes, the levels of the WAT markers pantothenate kinase 3 (Pank3), chemerin, and angiotensinogen (Agt) were up-regulated, whereas fatty acid-binding protein 4 (Fabp4) levels were down-regulated (Fig. 1*G*). Moreover, the master regulator of brown adipose tissue markers PR domain-containing 16 (PRDM16) was down-regulated (Fig. 1*G*). We also found that peroxisome proliferator-activated receptor γ coactivator 1α (PGC1α) was down-regulated in SIRT1-silenced adipocytes (Fig. 7, *A* and *F*). These results strongly suggest that SIRT1 reduction leads to physiologically dysfunctional adipocytes.

SIRT1 has been shown to regulate adipose tissue inflammation (30). We investigated the expression levels of the pro-inflammatory cytokine Tnf-α and anti-inflammatory cytokine IL-10 in SIRT1-silenced adipocytes. Interestingly, we found Tnf-α to be up-regulated, whereas IL-10 was down-regulated in SIRT1-silenced adipocytes compared with controls (Fig. 1*G*), suggesting that adipocytes are in an inflamed state when SIRT1 is reduced. Taken together, these findings show that 3T3-L1 adipocytes are not only hyperplastic but also smaller in size, dysfunctional, and inflamed when SIRT1 is reduced.

##### 3T3-L1 Preadipocytes Are Smaller in Size, Exhibit Enhanced Proliferation, and Escape Contact Inhibition When SIRT1 Is Knocked Down

**FIGURE 2.**
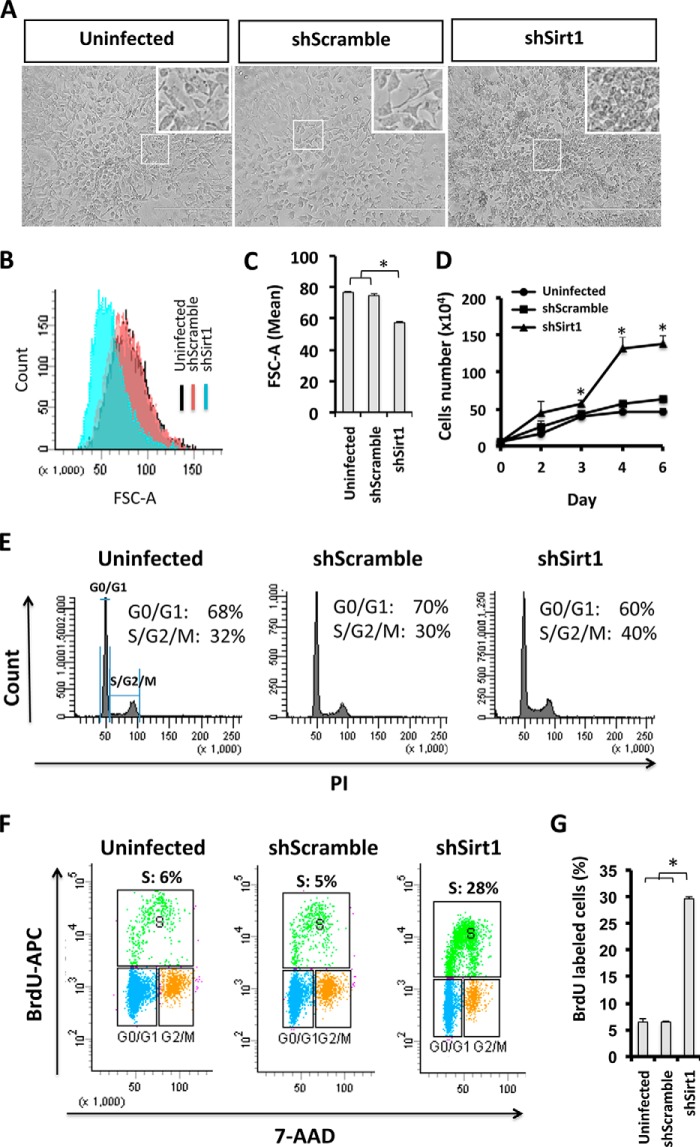
**SIRT1 knockdown increases the cell proliferation potential in 3T3-L1 preadipocytes.** *A*, microscopic phase-contrast images of 3T3-L1 cells for the indicated conditions. *Areas in boxes* are enlarged 6-fold at the *top right. Scale bars* = 400 μm. *B* and *C*, cell size was assessed by flow cytometric analysis. *Curves* represent cell number *versus* cell size FSC-A (forward scatter) and are quantified in *C*. Results are presented as mean ± S.E. from three independent experiments using Student's *t* test. *, *p* < 0.05. *D*, growth curve representing the number of 3T3-L1 cells of the indicated conditions at different time points (days 0, 2, 3, 4, and 6). Results are presented as mean ± S.E. from three independent experiments using Student's *t* test. *, *p* < 0.05. *E*, cell cycle analysis by flow cytometry. 3T3-L1 cells for the indicated conditions were stained with propidium iodide nuclear dye. Cellular DNA contents were determined by flow cytometric analysis, and cells were distributed in three phases of the cycle (G_0_/G_1_, S, and G_2_/M). The S and G_2_/M phases were combined to represent 2n-fold DNA content and compared with G_0_/G_1_ (1n-fold DNA content). Results are presented in percent for each plot and are representative of three independent experiments. *F*, flow cytometric analysis of BrdU incorporation in 3T3-L1 cells. Cells were stained with BrdU-APC antibodies and 7-AAD nuclear dye, and S phase (*green population*) was defined as BrdU-APC-positive cells. *PI*, propidium iodide. *G*, quantification of BrdU incorporation presented in *F*. Results are presented as mean ± S.E. from three independent experiments using Student's *t* test. *, *p* < 0.05.

To assess whether the observed adipocyte hyperplastic phenotype is due to a dysregulation in cellular growth and cell cycle control in 3T3-L1 preadipocytes, we investigated the function of SIRT1 in preadipocyte proliferation. Using phase-contrast microscopy, we found that SIRT1-silenced 3T3-L1 preadipocytes exhibit a crowded cell phenotype that could indicate lack of cell contact inhibition (Fig. 2*A*). In addition, SIRT1-silenced 3T3-L1 cells were smaller in size compared with control cells, as confirmed by flow cytometry (Fig. 2, *B* and *C*). The mean FSC-A scatter was reduced significantly in SIRT1-silenced cells (57.33 ± 0.66 FSC-A) compared with control cells (shScrambled, 74.66 ± 0.88 FSC-A; uninfected, 76.66 ± 0.88 FSC-A). SIRT1-silenced 3T3-L1 preadipocytes also proliferated faster than control cells (Fig. 2*D*). The growth of control cells slowed down and plateaued with increasing confluence (3 days after plating) in contrast to SIRT1-silenced cells, which continued to proliferate. Therefore, in the absence of SIRT1, preadipocytes seem to escape contact inhibition compared with control cells.

We then analyzed the cell cycle of SIRT1-silenced cells by flow cytometry in cells growing in the linear log phase (60% confluent). Interestingly, we found that SIRT1-silenced cells have a higher percentage of cells in the S/G_2_/M cell cycle phases (40%) than shScramble and uninfected cells (30% and 32% respectively), as shown in Fig. 2*E*. To further verify enhanced cell proliferation potential in 3T3-L1 preadipocytes upon SIRT1 silencing, we assessed BrdU incorporation in S phase using flow cytometry (Fig. 2, *F* and *G*). Confluent SIRT1-silenced cells showed a significantly higher DNA incorporation (28%) compared with control cells (5% and 6% for shScramble and uninfected cells, respectively). Overall, we conclude that SIRT1 reduction in 3T3-L1 preadipocytes results in an increased cell division rate, small cell size, and a bypass of contact inhibition, all indicative of dysregulation in cell cycle control.

##### Quantitative Proteomics and Pathway Analysis of 3T3-L1 Preadipocytes with Silenced SIRT1 Identifies Hundreds of Differentially Regulated Proteins and Uncovers Altered Metabolic Signaling Pathways

To explore the pathways that promote the SIRT1 knockdown-induced cell proliferation phenotype in 3T3-L1 preadipocytes, we employed mass spectrometry-based proteomics analysis using SILAC (24, 31). As a strategy, we used a common internal standard for quantitation to differentially characterize the expression of proteins isolated from shSirt1, shScramble, and uninfected 3T3-L1 preadipocytes.

**FIGURE 3.**
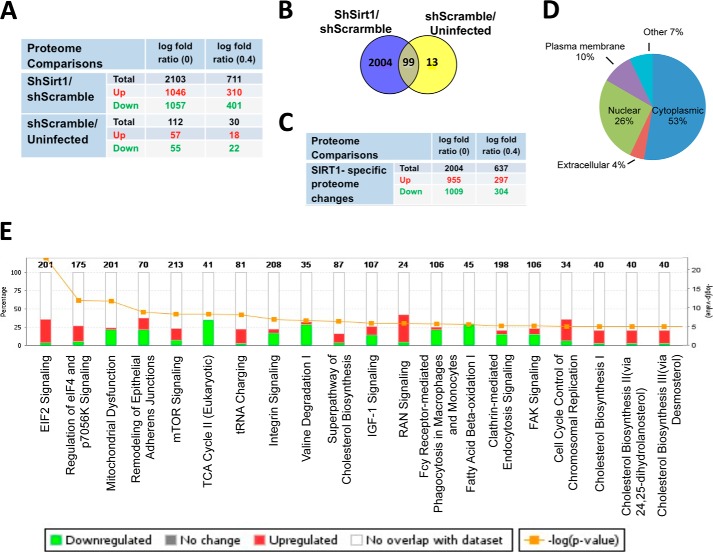
**Proteomics and pathway analyses of SIRT1-silenced 3T3-L1 preadipocytes.** *A*, the number of statistically significant proteome changes in shSirt1 *versus* shScramble and shScramble *versus* uninfected preadipocytes proteome comparisons at the indicated cutoff value of log_2_ -fold changes. Highlighted in *red* and *green* are the numbers of up-regulated and down-regulated gene products, respectively. *B*, overlap of gene products between shSirt1/shScramble and shScramble/uninfected proteome comparisons. *C*, the number of statistically significant SIRT1-specific proteome changes at the indicated cutoff value of log_2_ -fold changes. Highlighted in *red* and *green* are the numbers of up-regulated and down-regulated gene products, respectively. *D*, percentages of gene annotation by subcellular localization as indicated in shSirt1/shScramble proteome comparison. *E*, IPA prediction of the top 20 affected canonical pathways of SIRT1-specific proteome changes in the shSirt1/shScramble proteome analysis. The axes show percentages of overlapped genes in each pathway and the *p* value of overlap (IPA-generated). Highlighted in *red* and *green* is the percentage of up-regulated and down-regulated gene products in each pathway, respectively.

Analysis of significant proteome changes of the shSirt1/shScramble proteome using Limma (28) revealed 2103 differentially expressed proteins (false discovery rate cutoff, 0.05; Fig. 3*A*). 1046 proteins were identified as up-regulated and 1057 as down-regulated. Further filtration of a minimum log_2_ -fold change of 0.4 reduced the set to 711 differentially expressed proteins, of which 310 were up-regulated and 401 were down-regulated.

An independent pairwise comparison of the proteomes of the shScramble and uninfected controls using Student's *t* test and correction for multiple hypothesis testing according to Benjamini-Hochberg revealed 112 proteins as differentially expressed between the control sets when requiring a false discovery rate of 0.05 or less (Fig. 3*A*). These 112 products were excluded from downstream pathway analysis because they represent changes because of viral infection and are not specific to SIRT1 silencing. After excluding shScramble-specific proteome, we obtained a list of 2004 shSirt1-specific and significant proteome changes (Fig. 3, *B* and *C*), which were employed in downstream pathway analysis to identify altered signaling pathways in shSirt1 compared with the shScramble proteome.

To explore molecular and cellular pathways altered by SIRT1 knockdown, we subjected the 2004 SIRT1-specific proteome changes to IPA. With respect to subcellular localization, the following categories were annotated: 53% cytoplasmic, 26% nuclear, 10% plasma membrane, 4% extracellular, and the rest unannotated (Fig. 3*D*). The top 20 affected canonical pathways include transcription regulatory pathways (EIF2 and EIF4 signaling), metabolic pathways (mitochondrial dysfunction, TCA cycle, amino acids, fatty acid β-oxidation pathways, and cholesterol biosynthesis pathways), and cell cycle control of the chromosome replication pathway, among others (Fig. 3*E*). Consistent with known metabolic functions of SIRT1, the mitochondrion biogenesis, TCA cycle, and fatty acid β-oxidation pathways were all inhibited upon SIRT1 silencing. Cell cycle regulation, DNA replication, and EIF2, EIF4, and RAs related nuclear protein (RAN) pathways represent prominent pathways activated by SIRT1 knockdown (Fig. 3*E*). This proteomics analysis has uncovered differentially affected pathways that are consistent with well characterized SIRT1 metabolic functions and reflect the enhanced proliferation phenotype described above for SIRT1-silenced 3T3-L1 preadipocytes.

##### Focused Pathway Analysis of Proteome Changes Induced by SIRT1 Reduction Reveals Enrichment in Cell Cycle Regulatory Pathways and Uncovers c-Myc as the Most Affected Pathway

**FIGURE 4.**
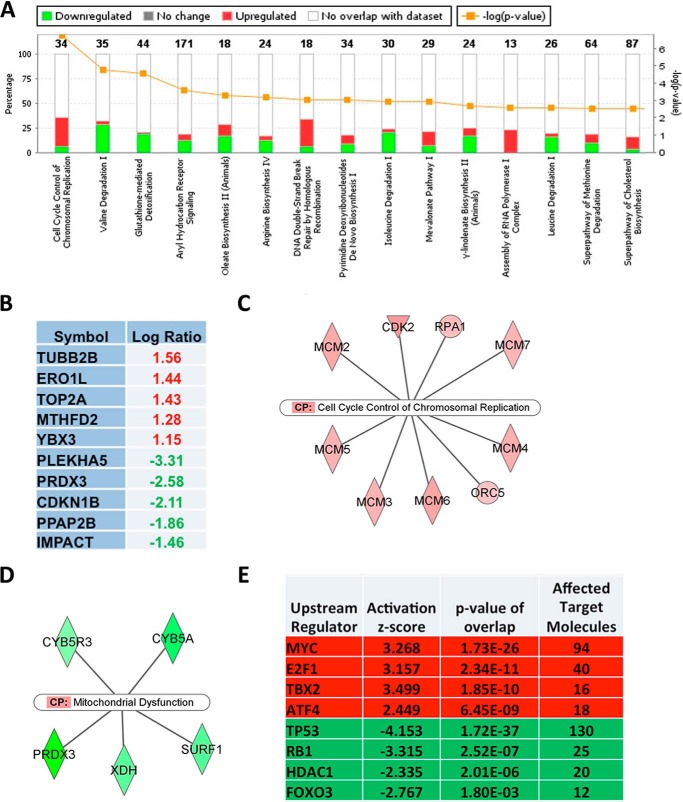
**Focused pathway analysis of proteome changes in SIRT1-silenced preadipocytes reveals enrichment in cell cycle regulatory pathways.** *A*, the top 15 IPA-predicted affected canonical pathways of SIRT1-specific proteome changes. The axes show percentages of overlapped genes in each pathway and the *p* value of overlap (IPA-generated). Highlighted in *red* and *green* is the percentage of up-regulated and down-regulated gene products in each pathway, respectively. *B*, the top five identified up-regulated and down-regulated gene products in the dataset as highlighted in *red* and *green*, respectively. *C*, the top nine affected molecules designated to the cell cycle control of chromosomal replication canonical pathway (*CP*). *D*, the top five affected molecules designated to the mitochondrial canonical pathway. *Green* shows down-regulated and *red* shows up-regulated genes products in the dataset. *E*, IPA predictions of affected regulators in SIRT1-specific proteome changes. Highlighted in *red* and *green* are pathways predicted as activated or inhibited, respectively. The ranking is on the basis of assigned IPA *p* values, and the degree of activation is on the basis of IPA assigned z scores of activation. A z score of above +2 signifies activation, whereas a z score below −2 implies inhibition.

To extend our pathway analysis and identify factors critical in mediating SIRT1 function in 3T3-L1 preadipocytes, we filtered our dataset using a value of 0.4 of log_2_ -fold change and obtained a focused set of 637 SIRT1-exclusive proteome changes (Fig. 3*C*), 297 of which were up-regulated and 304 of which were down-regulated to IPA. We subjected this focused set to an IPA analysis, which found cell cycle control of chromosomal replication as the most enriched canonical pathway (Fig. 4*A*). In addition, altered amino acid metabolism signaling pathways and DNA double-strand break repair by homologous recombination were also reported as activated.

Reflecting the observed phenotype, p27^CDKN1B^ and DNA topoisomerase 2α catalytic subunit p170 (TOP2A), gene products critical to cell cycle progression and DNA synthesis, were also expressed differentially (Fig. 4*B*). Although the protein level of the key cell cycle regulator p27 was reduced (log_2_ -fold ratio, −2.11), TOP2A was among the top three highly expressed gene products, which further supported the notion that SIRT1 serves in regulating cellular growth and chromosomal replication (Fig. 4*B*). Moreover, we identified a list of up-regulated gene products that were mapped by IPA to the cell cycle control of chromosomal replication canonical pathway (Fig. 4*C*). We also identified the down-regulated gene products that were mapped by IPA to the mitochondrial dysfunction canonical pathway (Fig. 4*D*). These proteomics findings show that SIRT1 reduction is linked to differential expression of cell cycle regulators that may drive the enhanced proliferation as well as dysregulation of mitochondrial factors, affecting the metabolic state of 3T3-L1 preadipocytes. The focused pathway analysis provides intriguing hints at what the molecular mechanisms behind SIRT1-specific proteome changes may be (Fig. 4*E*). We identified upstream regulators using the IPA software database of known pathway structures. With 94 c-Myc target molecules affected, c-Myc was predicted to be activated with a z-score of +3.26 and a *p* value of 1.73E-26. Besides c-Myc, the transcription factors E2F1, TBX2, and ATF4 were predicted activated upstream regulators, whereas p53, RB1, HDAC1, and FOXO3 were predicted to be inhibited. We found that the c-Myc pathway is the most activated pathway in SIRT1 knockdown 3T3-L1 preadipocytes and is potentially driving the observed enhanced proliferation phenotype.

##### Enhanced Proliferation in SIRT1 Knockdown Is Likely Driven by c-Myc Activation and Cell Cycle Regulators p27 and CDK2

**FIGURE 5.**
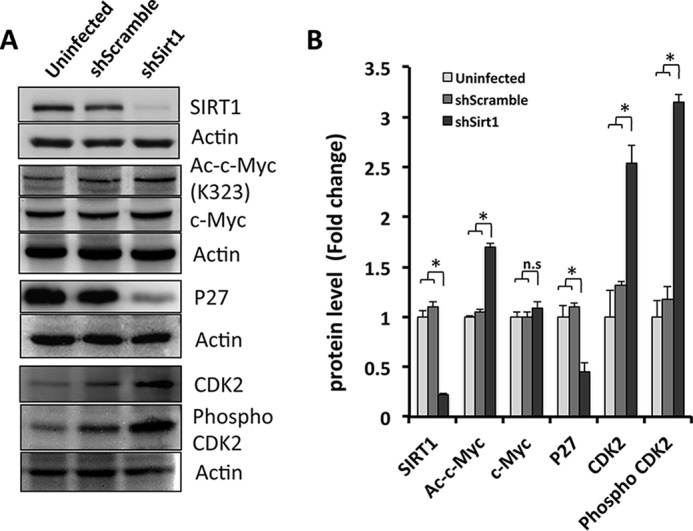
**c-Myc and cell cycle key player p27 and CDK2 expression and activation are altered in SIRT1 knockdown preadipocytes.** *A*, Western blotting was performed using antibodies against SIRT1, acetylated c-Myc (Lys-323), total c-Myc, total p27, total CDK2, phospho-CDK2, and actin as a loading control from cell lysates derived from 3T3-L1 cells for the indicated conditions. *B*, quantification of the data in *A*. Results are presented as mean ± S.E. from three independent experiments using Student's *t* test. *, *p* < 0.05; *n.s.*, not significant.

To confirm whether the highly proliferative phenotype in SIRT1 knockdown 3T3-L1 cells correlates with altered activities of key regulators uncovered by the proteomics screen, such as c-Myc, we determined the protein level and activity of c-Myc in SIRT1-silenced 3T3-L1 cells (Fig. 5, *A* and *B*). We found that SIRT1 protein levels were low in SIRT1-silenced cells compared with shScrambled cells and uninfected cells (0.22 compared with 1- and 1.1-fold, respectively). SIRT1 is known to regulate the activity of its target protein through posttranslational modifications, including deacetylation. Importantly, c-Myc acetylation at lysine 323, a known target residue for SIRT1 deacetylation reported in HeLa cells and 293T cells (32), was increased in SIRT1 knockdown cells (1.7-fold compared with shScrambled and uninfected cells with 1- and 1.1-fold, respectively). Total c-Myc expression appeared not to be changed significantly. This suggests that, in the absence of SIRT1, c-Myc is acetylated differentially, altering its biological activity.

To further validate our proteomics findings, we monitored the total p27 protein levels as well as the total and phosphorylated CDK2 protein levels. Interestingly, SIRT1 knockdown led to reduced p27 (0.4-fold). Both CDK2 and phospho-CDK2 were increased to more than 2-fold compared with control shScrambled and uninfected cells (Fig. 5, *A* and *B*). Overall, these data suggest that SIRT1 seems to control c-Myc activity and growth of proliferating preadipocytes.

##### Enhanced MCE of Differentiating Adipocytes in SIRT1 Knockdown

**FIGURE 6.**
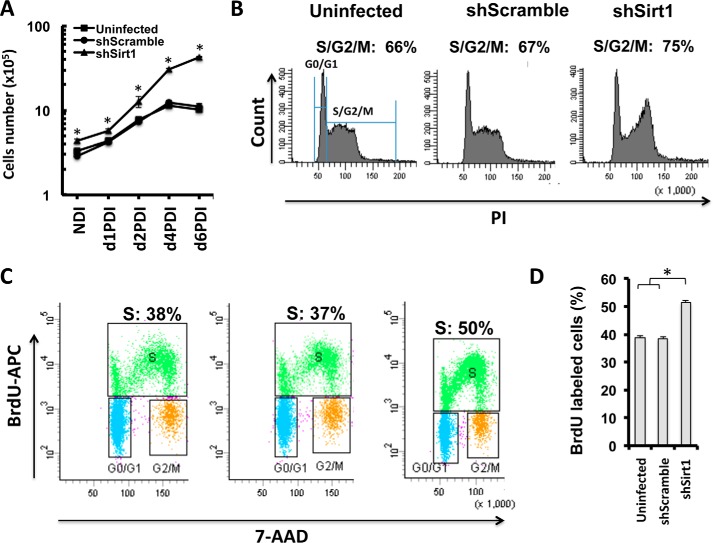
**SIRT1 knockdown affects MCE of differentiating adipocytes.** *A*, growth curve for differentiating 3T3-L1 cells of the indicated conditions in a time course experiment with and without differentiation induction (no DI (*NDI*), d1PDI, d2PDI, d4PDI, and d6PDI). Results are representative as mean ± S.E. of three independent experiments using Student's *t* test. *, *p* < 0.05. *B*, cell cycle analysis by flow cytometry. 3T3-L1 cells of the indicated conditions were stained with propidium iodide (*PI*) on d1PDI. Cellular DNA contents were determined by flow cytometric analysis. G_0_/G_1_ represent 1n-fold DNA content and S and G_2_/M phases represent 2n-fold DNA content. Results are presented in percent for each plot. Results are representative of four independent experiments. *C*, flow cytometric analysis of BrdU incorporation for the indicated conditions of 3T3-L1 cells on d1PDI. Cells were stained with BrdU-APC and 7-AAD, and S phase (*green population*) was defined as BrdU-APC-positive cells. *D*, quantification of BrdU incorporation presented in *C*. Results are presented as mean ± S.E. from three independent experiments using Student's *t* test. *, *p* < 0.05.

To assess whether the observed hyperplastic phenotype is related to an increase in MCE potential in 3T3-L1 preadipocytes, we generated growth curves for SIRT1-silenced cells and control cells by cell counting at different time points after differentiation induction (Fig. 6*A*). This analysis revealed higher proliferation for SIRT1-silenced adipocytes compared with control cells between d1PDI and d2PDI, a time interval when cells are undergoing MCE (4) (Fig. 6*A*). Control cells stopped dividing and plateaued at d4PDI when they exited the cell cycle to differentiate into adipocytes. However, SIRT1-silenced cells continued to divide after d4PDI, indicating that MCE exit is delayed compared with control cells (Fig. 6*A*).

To further confirm the MCE phenotype, we analyzed the cell cycle of SIRT1-silenced cells during MCE at d1PDI by flow cytometry. A higher proportion of SIRT1-silenced cells was observed in S/G_2_/M cell cycle phases compared with control cells (75% for shSirt1 compared with 67% and 66% for shScramble and uninfected cells, respectively (Fig. 6*B*). By assessing BrdU incorporation as a measure of proliferative potential during the MCE stage, we additionally found that differentiating SIRT1-silenced cells incorporated significantly more BrdU (50%) compared with controls shScramble and uninfected cells (37% and 38%, respectively), as shown in Fig. 6, *C* and *D*. These results demonstrate that SIRT1-silenced cells exhibit elevated MCE and delayed MCE exit phenotypes.

##### SIRT1 Reduction Dysregulates MCE and Adipogenic Regulators in Differentiating 3T3-L1 Adipocytes

**FIGURE 7.**
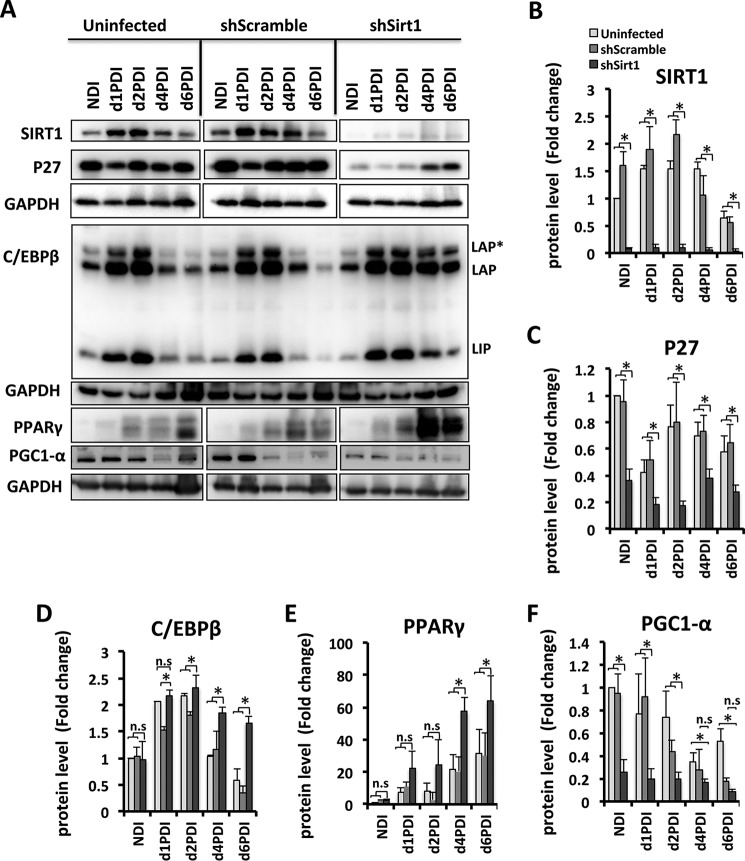
**SIRT1 reduction leads to dysregulation of MCE and adipogenic factors in differentiating 3T3-L1 adipocytes.** *A*, Western blot analyses were performed for cell lysates of 3T3-L1 cells for the indicated conditions using antibodies against SIRT1, p27, C/EBPβ, PPARγ, and PGC1α. GAPDH was used as a loading control. Equal amounts of proteins lysates were run in separate gels. Membranes were incubated together with antibodies and developed at the same time with ECL substrate, and pictures were taken with the same intensity. Results are representative of three independent experiments. *NDI*, no DI; *LAP*, liver enriched activator protein; *, full length; *LIP*, liver enriched inhibitor proteins are C/EBP-β isoforms. *B–F*, Western blot bands were quantified and normalized to the GAPDH control. Results are represented as mean ± S.E. of three independent experiments using Student's *t* test. *, *p* < 0.05; *n.s.*, not significant.

Protein levels of SIRT1, p27, C/EBPβ, and PPARγ in shSirt1 3T3-L1 preadipocytes undergoing differentiation were evaluated over time (Fig. 7). SIRT1 levels were significantly decreased at all differentiation time points in SIRT1-silenced cells compared with controls (Fig. 7, *A* and *B*). Importantly, SIRT1 expression was increased in control cells at early differentiation stages (d1PDI to d2PDI) but then decreased at late differentiation stages (d4PDI to d6PDI), which suggests that SIRT1 regulates the MCE phase of differentiation.

A previous study showed that p27 levels are decreased during MCE of differentiating 3T3-L1 cells (33), but how SIRT1 regulates p27 levels during differentiation is not known. We therefore analyzed p27 expression during differentiation of SIRT1-silenced 3T3-L1 cells. We found that, in control cells, the expression profile of p27 during differentiation is consistent with previous reports (33). p27 levels are reduced in early MCE (d1PDI) and subsequently increase to allow mitotic exit (d2PDI-d4PDI) (Fig. 7*A*). However, in SIRT1-silenced cells, we observed a significant down-regulation of p27 protein expression compared with controls at d1PDI to d6PDI (Fig. 7, *A* and *C*), which correlates with the observed enhanced MCE proliferation and delayed MCE exit.

Protein expression of C/EBPβ is known to be required for MCE during adipogenesis (34). Therefore, we evaluated its expression in SIRT1-silenced cells and found that it was up-regulated significantly compared with controls, both during MCE (d1PDI and d2PDI) and the late stages of differentiation (d4PDI and d6PDI) (Fig. 7, *A* and *D*). These data indicate that SIRT1 knockdown affects C/EBPβ expression to induce enhanced MCE and to delay MCE exit.

Finally, we analyzed the protein expression level of adipogenic factors in SIRT1-silenced cells during differentiation. PPARγ is a well known and studied metabolic target of SIRT1 and functions as a master regulator for adipocyte differentiation (35, 36). Therefore, adequate regulation of PPARγ by SIRT1 is necessary to control efficient adipogenesis. We also found PPARγ to be increased significantly at d4PDI and d6PDI in SIRT1-silenced cells compared with control cells (Fig. 7, *A* and *E*), which is in line with what was has been shown previously (19). Together, these observations support the importance of SIRT1 as a key player in the regulation of both MCE and adipogenic regulation.

##### SIRT1 Reduction Drives Enhanced MCE Proliferation via c-Myc Activation in Differentiating Adipocytes

**FIGURE 8.**
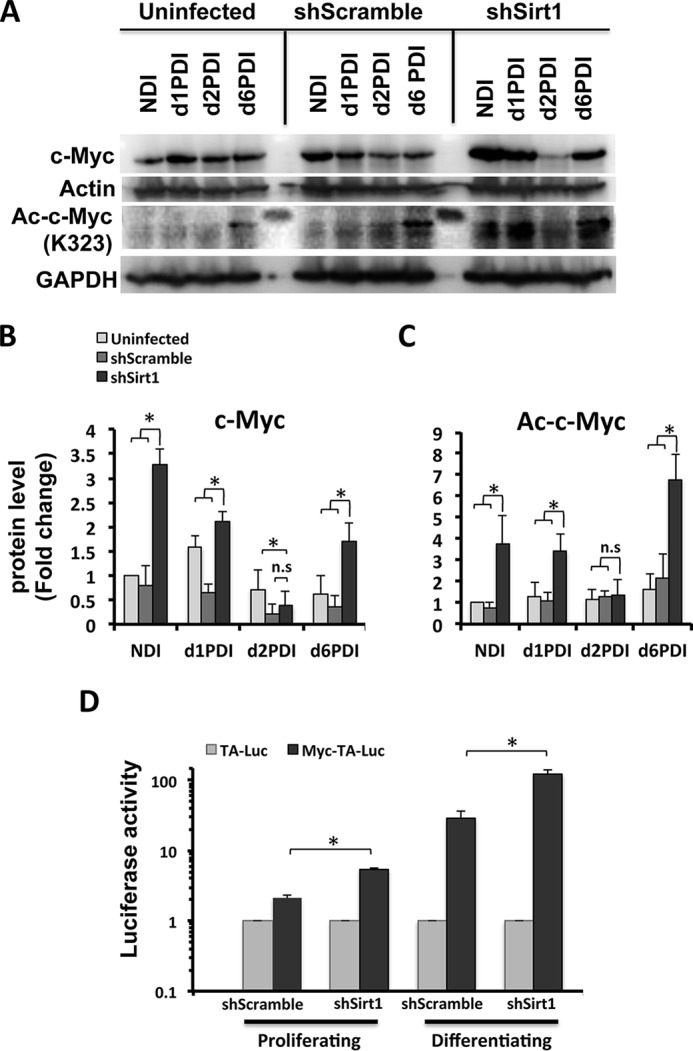
**c-Myc expression and activation are altered, whereas SIRT1 is reduced during MCE of differentiating 3T3-L1 adipocytes.** *A*, Western blot analysis was performed from cell lysates of 3T3-L1 cells for the indicated conditions using antibodies against total c-Myc and acetylated c-Myc (Lys-323). Actin and GAPDH were used as loading controls. Results are representative of three independent experiments. *NDI*, no DI. *B–C*, Western blot bands were quantified and normalized to the loading control. *D*, assessment of c-Myc transcriptional activity using a luciferase reporter assay. shSirt1 3T3-L1 cells were transfected with either pMyc-TA-Luc or pTA-Luc plasmids combined with pSV-β-gal plasmids, and, the following day, cells were induced to differentiate. Luciferase activity was measured and normalized to β-gal expression for both proliferating cells and differentiating cells on d1PDI. The luciferase activity for shSirt1 cells was compared with control shScramble control cells. Results are represented as mean ± S.E. of three independent experiments using Student's *t* test. *, *p* < 0.05; *n.s.*, not significant.

Following up on our observations in the proteomics screen on protein expression differences induced by SIRT1 knockdown and the subsequent characterization of c-Myc, we revisited those potential key players in SIRT1-mediated cell cycle control during differentiation of SIRT1-silenced adipocytes. Total c-Myc protein as well as its acetylation status (known SIRT1 deacetylation substrate Lys-323) was analyzed in shSirt1, shScrambled, and uninfected 3T3-L1 cells undergoing differentiation at different time points. Interestingly, c-Myc levels were increased significantly in confluent cells with no differentiation induction and in differentiating cells at d1PDI and d6PDI compared with controls (Fig. 8, *A* and *B*). Moreover, c-Myc was found to be hyperacetylated in nondifferentiating and differentiating SIRT1 knockdown cells when compared to controls (Fig. 8, *A* and *C*). No significant difference was found for total and the acetylated c-Myc on d2PDI between SIRT1-silenced adipocytes and controls (Fig. 8, *A–C*). Therefore, during SIRT1 knockdown, c-Myc appears not only to be up-regulated but also hyperacetylated, driving enhanced MCE proliferation and differentiation.

To further characterize the mechanism of SIRT1 in driving hyperplasia, we assessed the c-Myc transcriptional activity in both proliferating and differentiating SIRT1 knockdown preadipocytes. We performed a luciferase reporter assay where we measured luciferase expression under c-Myc regulatory response elements. We found elevated c-Myc transcriptional activity in both proliferating and differentiating SIRT1-silenced cells compared with control cells (Fig. 8*D*). These results suggest that SIRT1 suppresses c-Myc activity. Therefore, c-Myc seems to be a strong candidate for a driver of enhanced proliferation and MCE, leading to a dysfunctional hyperplastic adipocyte phenotype in the absence of SIRT1. Taken together, we conclude that the role of SIRT1 in regulating the proliferation of preadipocyte and MCE differentiation is mediated through c-Myc activation.

**FIGURE 9.**
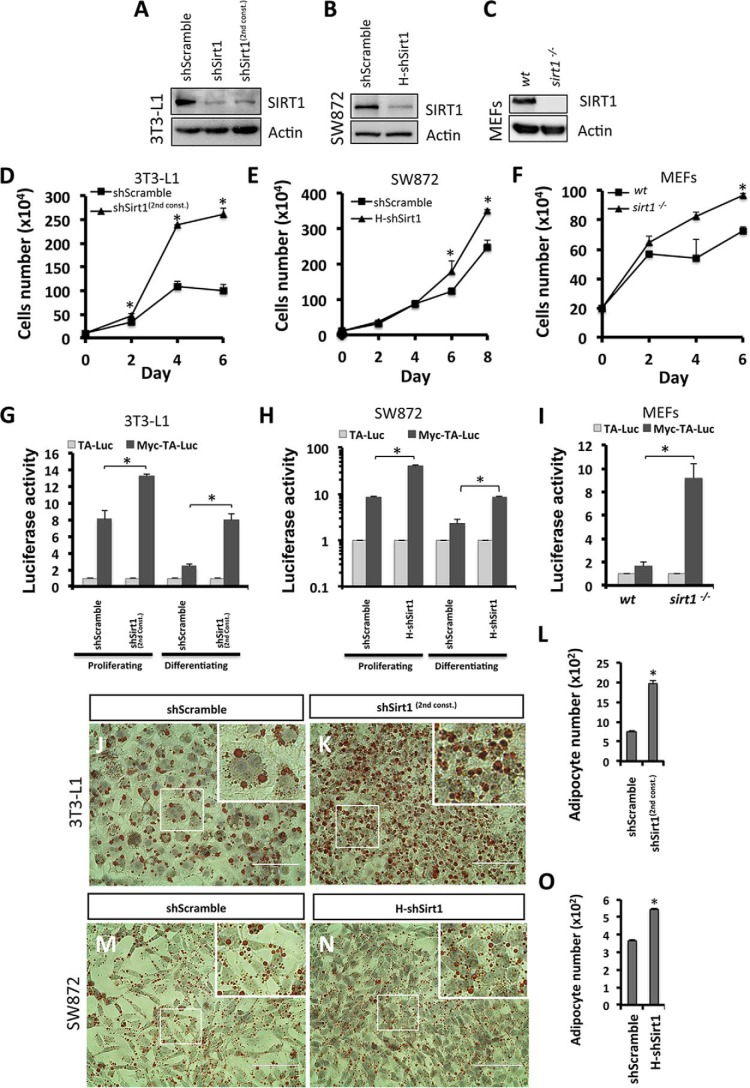
**SIRT1 knockdown and c-Myc activation drive the enhanced proliferation phenotype in different cell types.** *A–C*, Western blotting was performed using antibodies against SIRT1 for the indicated conditions of 3T3-L1 preadipocytes: two different shSirt1 constructs (*A*), SW872 preadipocytes (shScramble and H-shSirt1 lentivirus-infected cells, *B*), and MEF cells (WT and *sirt1*^−/−^, *C*). Actin was used as a loading control. *D–F*, growth curves representing cell numbers of 3T3-L1 (*D*), SW872 cells (*E*), and MEF cells (*F*) of the indicated conditions at different time points. Results are representative as mean ± S.E. of three independent experiments using Student's *t* test. *, *p* < 0.05. *G* and *I*, assessment of c-Myc transcriptional activity using a luciferase reporter assay in shSirt1^(2nd const.)^ 3T3-L1 cells (*G*), H-shSirt1 SW872 cells (*H*), and *sirt1*^−/−^ MEFs (*H*). Cells were transfected with either pMyc-TA-Luc or pTA-Luc plasmids combined with the pSV-β-gal plasmid. The following day, 3T3-L1 and SW872 cells were induced to differentiate. Luciferase activity was measured and normalized to β-gal expression for both proliferating cells and differentiating cells on d1PDI (3T3-L1 cells) and d4PDI (SW872 cells). Luciferase activity for shSirt1^(2nd const.)^ cells and H-shSirt1 cells was compared with the respective shScramble control cells and that of *sirt1*^−/−^ MEF cells with WT MEF control cells. Results are represented as mean ± S.E. of three independent experiments using Student's *t* test. *, *p* < 0.05. *J–O*, Oil Red O staining of differentiated 3T3-L1 adipocytes (*J* and *K*) and SW872 adipocytes (*M* and *N*) for the indicated conditions. Nuclei were stained with hematoxylin. The *areas in boxes* are enlarged 4-fold at the *top right. Scale bars* = 100 μm. *L* and *O*, quantification of the data in *J–K* and *M* and *N*, respectively, for the number of adipocytes for each condition. Results are presented as mean ± S.E. from six different fields of three independent experiments using Student's *t* test. *, *p* < 0.05.

To determine whether the enhanced proliferation phenotype driven by c-Myc activation in SIRT1 knockdown cells is not an shRNA off-target effect, we used an independent Sirt1 shRNA in 3T3-L1 cells, indicated as shSirt1^(2nd const.)^. We show a similar reduction of SIRT1 protein in both constructs (Fig. 9*A*). Growth curve analysis of proliferating shSirt1^(2nd const.)^ 3T3-L1 preadipocytes shows enhanced proliferation at different time points compared with control cells (Fig. 9*D*). Moreover, elevated c-Myc transcriptional activity was validated to be elevated in shSirt1^(2nd const.)^ compared with control cells under both proliferating and differentiating conditions (Fig. 9*G*). To further show that this phenotype is not 3T3-L1 cell type-specific, we used human SW872 preadipocytes to silence SIRT1 (indicated as H-shSirt1) in addition to *sirt1*^−/−^ MEF cell lines (Fig. 9, *B* and *C*). Similarly, we observed enhanced proliferation and elevated c-Myc transcriptional activity in H-shSirt1 SW872 cells (Fig. 9, *E* and *H*, respectively) and in *sirt1*^−/−^ MEFs (Fig. 9, *F* and *I*, respectively) compared with their respective controls. Furthermore, to determine whether SIRT1 reduction drives adipocyte hyperplasia in 3T3-L1 adipocytes using an independent shRNA and SW872 adipocytes using H-shSirt1, preadipocytes were induced to differentiate, and mature adipocytes were fixed and stained with Oil Red O on d6PDI and d10PDI of 3T3-L1 (Fig. 9, *J* and *K*) and SW872 cells (Fig. 9, *M* and *N*), respectively. Our data illustrate that both differentiated SIRT1-silenced 3T3-L1 and SW872 cells were significantly higher in number than control cells (1976 ± 85 adipocytes/mm^2^ in shSirt1^(2nd const.)^ *versus* 742 ± 42 adipocytes/mm^2^ in shScramble and 544 ± 7 adipocytes/mm^2^ in H-shSirt1 *versus* 367 ± 6 adipocytes/mm^2^ in shScramble, respectively) (Fig. 9, *L* and *O*). Taken together, these findings reveal that c-Myc activation driven by SIRT1 reduction causes a hyperplastic phenotype in other adipocyte cellular models.

##### The Adipocyte Hyperplasia Phenotype Does Not Develop When Both the SIRT1 and c-Myc Protein Levels Are Reduced

**FIGURE 10.**
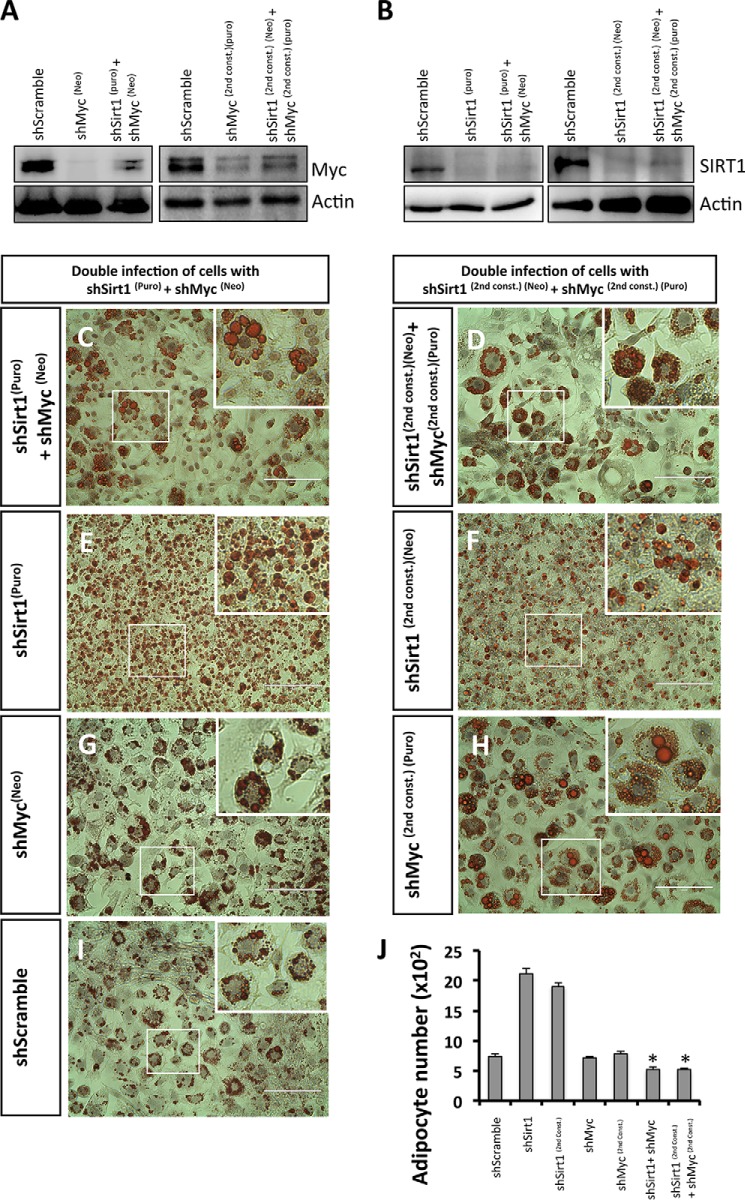
**c-Myc reduction prevents the SIRT1 knockdown-induced adipocyte hyperplasia phenotype.** *A* and *B*, Western blotting was performed using antibodies against c-Myc (*A*) and SIRT1 (*B*) for the following conditions of 3T3-L1 preadipocytes: control shScramble, two different constructs for shMyc (*A*), shSirt1 (*B*), and two different combinations of double infection using different antibiotics selections (puromycin (*puro*) and neomycin (*neo*)) (*A* and *B*). *C–I*, Oil Red O staining of differentiated 3T3-L1 adipocytes for the indicated conditions. Nuclei were stained with hematoxylin. The *areas in boxes* are enlarged 4-fold at the *top right. Scale bars* = 100 μm. *J*, quantification of the data in *C–I* for the number of adipocytes for each condition. Results are presented as mean ± S.E. from six different fields of three independent experiments using Student's *t* test. *, *p* < 0.05.

To directly test the relevance of c-Myc in the hyperplasia phenotypes associated with SIRT1 reduction, we simultaneously knocked down both SIRT1 and c-Myc in 3T3-L1 preadipocytes using independent shRNA constructs (Fig. 10). We selected double-infected cells using two antibiotics (puromicyn and neomycin) and confirmed knockdown by Western blot analysis using antibodies against c-Myc or SIRT1 (Fig. 10, *A* and *B*, respectively). Remarkably, when analyzing differentiated adipocytes using Oil Red O staining and cell counting, we found that cells silenced simultaneously with SIRT1 and c-Myc did not develop the hyperplastic phenotype (514 ± 42 adipocytes/mm^2^ in shSirt1 + shMyc and 509 ± 41 adipocytes/mm^2^ in shSirt1^(2nd const.)^ + shMyc^(2nd const.)^) (Fig. 10, *C*, *D*, and *J*) compared with control shSirt1 (2123 ± 83 adipocytes/mm^2^ in shSirt1 and 1909 ± 50 adipocytes/mm^2^ in shSirt1^(2nd const.)^) (Fig. 10, *E*, *F*, and *J*) or shScramble (742 ± 37 adipocytes/mm^2^ in shScramble) (Fig. 10, *I* and *J*). The shSirt1 + shMyc cells appeared similar in size to shScramble shRNA-infected adipocytes but not to shSirt1 adipocytes (Fig. 10, *C–F* and *I*). We did not observe any phenotypic difference by Oil Red O staining of shMyc-silenced adipocytes compared with shScramble control adipocytes (Fig. 10, *G* and *H–J*). These results suggest that c-Myc knockdown prevents the development of the hyperplastic phenotype induced by SIRT1 knockdown. We conclude that SIRT1 controls adipocyte hyperplasia in a c-Myc-dependent manner.

## Discussion

**FIGURE 11.**
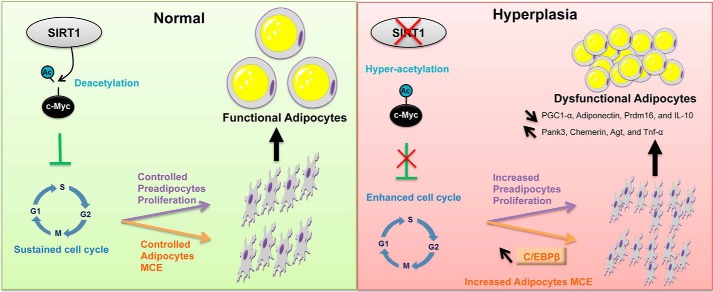
**Model of SIRT1 regulation of preadipocyte proliferation and adipocyte MCE and differentiation.** The schematic illustrates how SIRT1 controls preadipocyte proliferation and differentiation through the c-Myc pathway. Reduction or absence of SIRT1 leads to hyperplastic adipogenesis (*right panel*), and the presence of SIRT1 mediates normal adipogenesis (*left panel*). *Arrow pointing top left*, increase; *arrow pointing bottom right*, decrease; ×, reduction or inhibition.

In this study, we describe a novel function of SIRT1 in curbing adipocyte hyperplasia. We show that SIRT1 knockdown results in hyperplastic, small, and inflamed adipocytes that appear to be dysfunctional metabolically and physiologically. We provide converging cellular, molecular, and proteomics evidence that SIRT1 is a critical regulator of proliferation in preadipocyte as well as MCE and differentiation in adipocytes. We identified pathways with potentially altered signaling upon SIRT1 silencing and, therefore, provide a hypothesis for the mechanism underlying the associated phenotypes of dysregulated cell proliferation and adipocyte hyperplasia *in vitro*. Importantly, we find that the c-Myc pathway is the most significantly activated pathway in SIRT1-silenced preadipocytes. As illustrated in the proposed model, under conditions of normally expressed SIRT1 (Fig. 11), SIRT1 controls the cell cycle of dividing preadipocytes and the MCE of differentiating adipocytes via regulation of c-Myc. These lead to the generation of a controlled number of functional adipocytes. However, when SIRT1 levels are reduced (Fig. 11), c-Myc becomes hyperacetylated, which leads to higher preadipocyte proliferation potential and enhanced adipocyte MCE during differentiation, which ultimately results in dysfunctional hyperplastic adipocytes.

Adipocyte hyperplasia has been suggested to result from overnutrition causing excessive commitment of mesenchymal stem cells to preadipocytes (4). Mice lacking p27, p21, or both exhibited increased fat pad mass because of adipocyte hyperplasia and metabolic dysregulation (37). For some genetic mouse models, adipocyte hyperplasia in the subcutaneous depot has been linked to increased insulin sensitivity (reviewed in Ref. 7). In a recent report, Wang *et al.* (38) employed an animal model to track *in vivo* adipogenesis under a high-fat diet and showed that adipocyte hypertrophy increased in all fat depots initially and was followed by enhanced hyperplasia in the visceral fat depot of male mice. The metabolic profile of hyperplastic visceral adipose tissue expansion has not yet been determined. It is likely that the outcome of adipocyte hyperplasia and the resulting complications of obesity may depend on the genetic background, gender differences, and the location of the fat depot hyperplasia (6, 10). Although it remains to be determined *in vivo*, we suggest that SIRT1 inactivation could participate in visceral adipose tissue depot hyperplasia and, possibly, add to the complications of visceral adipose tissue depot expansion and insulin resistance.

Although we are beginning to analyze the molecular mechanisms by which altered protein levels of SIRT1 may drive adipocyte hyperplasia, knowledge about the molecular mechanisms underpinning fat hyperplasia *in vivo* is lacking. Therefore, future work should include an analysis of the function of SIRT1/c-Myc in driving adipocyte hyperplasia in a possibly fat depot-dependent manner in obese subjects. c-Myc has been shown previously to induce gene expression of SIRT1, yet it is a direct SIRT1 target for deacetylation and subsequent destabilization (32). This leads to a reduction in c-Myc expression and the generation of a negative feedback loop that controls c-Myc-induced cellular transformation (32, 39). In this study, SIRT1 reduction in preadipocytes led to hyperacetylation of c-Myc, which may drive uncontrolled cell proliferation and, as a consequence, may generate hyperplastic dysfunctional adipocytes. The SIRT1/c-Myc axis appears to control the cell number and functional integrity of adipocytes.

The key cell cycle regulator p27 is a repression target of c-Myc (40). Interestingly, c-Myc has been shown to be among the earliest expressed genes during MCE of differentiating preadipocytes (33). We have shown that SIRT1 seems to control preadipocyte proliferation and adipocyte MCE through p27 regulation, a mechanism that likely involves c-Myc activity. It will be interesting to further investigate the functional interactions between SIRT1, c-Myc, and p27 in the regulation of adipocyte proliferation and differentiation.

Evidence from *in vitro* studies shows that a critical component in triggering MCE is the activation of the transcription factor C/EBPβ (4). C/EBPβ drives the expression of PPARγ andC/EBPα, which induces adipogenic gene expression, resulting in mitotic exit and the accumulation of mature adipocytes (4). By analyzing C/EBPβ expression during adipogenesis, we found that C/EBPβ was up-regulated during MCE and late differentiation in SIRT1-silenced adipocytes, which were found to be hyperplastic. These data suggest that SIRT1 controls MCE through C/EBPβ. Characterizing and understanding how and when SIRT1 regulates C/EBPβ expression during adipogenesis will help to better understand adipogenesis and adipocyte hyperplasia.

SIRT1 has been shown recently to be involved in brown remodeling of white adipose tissue (41). Whether SIRT1 reduction induces hyperplastic adipocytes that are altered physiologically remains to be explored. In our study, WAT tissue markers such as Pank3, Chemerin, and Agt are increased, whereas the brown adipose tissue master regulator Prdm16 is reduced in SIRT1-silenced adipocytes. These findings suggest that SIRT1 reduction led to physiologically dysfunctional adipocytes. Moreover, our analysis shows that the observed adipocyte hyperplastic phenotype resulting from silencing of SIRT1 is associated with a dysregulated metabolism and inflammation. Indeed, the expression levels of the metabolic markers PGC1-α and adiponectin are reduced, whereas the expression level of the proinflammatory cytokine Tnf-α is increased and that of the anti-inflammatory marker IL-10 is reduced.

Using an *in vitro* approach, here we uncover a role of SIRT1 in adipocyte hyperplasia. Our data suggest that the reduction of SIRT1 in preadipocytes induces the hyperacetylation of c-Myc, leading to an abnormal proliferation rate and differentiation into small, inflamed, and dysfunctional adipocytes. If the relevance of our findings is confirmed in humans, then a better understanding of the molecular mechanisms involved in SIRT1/c-Myc signaling pathways should lead to the development of new therapeutic strategies for obesity.

## Author Contributions

H. A. and N. A. M. developed the original concept and designed and oversaw the study. H. A., A. M., and A. H. carried out the experiments. M. A., N. G., A. M. B., and H. B. H. carried out the proteomics assays. J. G. oversaw the proteomics studies. M. S. B., D. A. S., and N. M. contributed to the sirt1 knockout experiments. J. P. contributed to the analysis of the flow cytometry assays. N. H. performed the statistical analysis of the proteomics data. R. D., M. E., and M. Z. S. contributed to the expression studies. H. A. and N. A. M. analyzed the data, assembled the figures, and wrote the manuscript. All authors read and approved the final manuscript.

## Footnotes

2 The abbreviations used are:

WAT: white adipose tissue
PPARγ: proliferator-activated receptor γ
MCE: mitotic clonal expansion
C/EBPβ: CCAAT/enhancer-binding protein β
MEF: mouse embryonic fibroblast
TA: TATA box
SILAC: stable isotope labeling by amino acids in cell culture
IPA: Ingenuity pathway analysis
FSC-A: forward scatter-A
d1PDI: day 1 post-differentiation induction
APC: allophycocyanin
DI: differentiation induction.

## Note added in proof.

There were several errors in Figs. 1 and 7*A* that were corrected in the proof. In Fig. 1, the actin immunoblot in Fig. 1*A* was a duplicate of the actin immunoblot featured in Fig. 5*A*. Also, the Oil Red shSIRT1 panel of Fig. 1*B* was duplicated in Fig. 10*E*. In Fig. 7*A*, the GAPDH controls for SIRT1 and P27 immunoblots were misordered, and the wrong GAPDH loading control for PGC1-a was inadvertently included. These errors have been corrected and do not affect the interpretations of results or conclusions of the paper.
